# A pilot cluster randomised controlled trial of a support and training intervention to improve the mental health of secondary school teachers and students – the WISE (Wellbeing in Secondary Education) study

**DOI:** 10.1186/s12889-016-3737-y

**Published:** 2016-10-06

**Authors:** Judi Kidger, Tracey Stone, Kate Tilling, Rowan Brockman, Rona Campbell, Tamsin Ford, William Hollingworth, Michael King, Ricardo Araya, David Gunnell

**Affiliations:** 1 School of Social and Community Medicine, University of Bristol, Canynge Hall, 39, Whatley Road, Bristol, BS8 2PS UK; 2 National Institute for Health Research Collaborations for Leadership in Applied Health Research and Care in the West (NIHR CLAHRC West), 9th Floor, Whitefriars, Lewins Mead, Bristol, BS1 2NT UK; 3 University of Exeter Medical School, South Cloisters, St Luke’s Campus, Exeter, EX1 2LU UK; 4 Division of Psychiatry (Faculty of Brain Sciences), University College London, 6th Floor, Maple House, 149, Tottenham Court Road, London, W1T 7NF UK; 5 Ricardo Araya, Professor of Global Mental Health, London School of Hygiene and Tropical Medicine, Keppel Street, London, WC1E 7HT UK

**Keywords:** Teacher mental health, Mental health in schools, Adolescence, Wellbeing, Pilot randomised controlled trial

## Abstract

**Background:**

Secondary school teachers are at heightened risk of psychological distress, which can lead to poor work performance, poor quality teacher-student relationships and mental illness. A pilot cluster randomised controlled trial (RCT) – the WISE study – evaluated the feasibility of a full-scale RCT of an intervention to support school staff’s own mental health, and train them in supporting student mental health.

**Methods:**

Six schools were randomised to an intervention or control group. In the intervention schools i) 8–9 staff received Mental Health First Aid (MHFA) training and became staff peer supporters, and ii) youth MHFA training was offered to the wider staff body. Control schools continued with usual practice. We used thematic qualitative data analysis and regression modelling to ascertain the feasibility, acceptability and potential usefulness of the intervention.

**Results:**

Thirteen training observations, 14 staff focus groups and 6 staff interviews were completed, and 438 staff (43.5 %) and 1,862 (56.3 %) students (years 8 and 9) completed questionnaires at baseline and one year later. MHFA training was considered relevant for schools, and trainees gained in knowledge, confidence in helping others, and awareness regarding their own mental health. Suggestions for reducing the length of the training and focusing on helping strategies were made. A peer support service was established in all intervention schools and was perceived to be helpful in supporting individuals in difficulty – for example through listening, and signposting to other services - and raising the profile of mental health at a whole school level. Barriers to use included lack of knowledge about the service, concerns about confidentiality and a preference for accessing support from pre-existing networks.

**Conclusions:**

The WISE intervention is feasible and acceptable to schools. Results support the development of a full-scale cluster RCT, if steps are taken to improve response rates and implement the suggested improvements to the intervention.

**Trial registration:**

International Standard Randomised Controlled Trial Number: ISRCTN13255300 retrospectively registered 28/09/16.

**Electronic supplementary material:**

The online version of this article (doi:10.1186/s12889-016-3737-y) contains supplementary material, which is available to authorized users.

## Background

Teachers have been found to be at relatively high risk of common mental health disorders such as depression and anxiety compared to other workers [1–4]. In a recent survey of 555 secondary school teachers covering 8 schools in the South West of England [5], mean wellbeing according to the Warwick Edinburgh Wellbeing Scale (WEMWBS) was approximately four points below the norm found for the general working population [6], and 19.4 % of the sample had moderate to severe depression, compared to a general population prevalence of 8–10 % [7–9]. Individuals who are stressed or depressed are less likely to be able to do their job effectively, either due to increased absence from work [10, 11], or reduced productivity whilst at work, commonly described as ‘presenteeism’ [10, 12]. In the case of teachers, this reduced productivity may have an impact on student learning, as teachers with poor mental health may find it difficult to manage classes effectively, and to develop supportive relationships with students [13]. Supportive teacher-student relationships are associated with higher student engagement and achievement, and predict lower student mental health problems in the future [14, 15]. Conversely, difficult teacher-student relationships have been linked to psychiatric disorder in the student, and exclusion from school three years later [16].

Reasons cited for teachers’ heightened risk of mental health problems include excessive workload, lack of autonomy, poor salary, perceived lack of status and pressure to ‘perform’ in a context in which schools are increasingly regulated and judged against an array of externally determined targets [17–20]. Further, a lack of support in the workplace may exacerbate such stresses: qualitative studies have highlighted a culture of teachers coping with challenging situations alone, and not feeling able to confide in colleagues when feeling stressed or depressed [21, 22]. Research also indicates that, although teachers are expected to provide appropriate support to vulnerable children including those with behavioural and mental health difficulties, for whom they are the most commonly contacted group [23], they are not given adequate support or training to do this effectively, and this can be a further source of workplace stress and distress [24].

There is therefore a need for interventions that both equip school staff to support vulnerable students better and that support staff’s own mental health. Better training for staff in working with students experiencing mental and emotional difficulties is likely to lead to improved staff-student relationships, which will potentially lead to favourable academic and health outcomes for students [16], and improvements in staff mental health via increased job satisfaction and decreased stress [5, 25]. Direct support for staff themselves is also likely to lead to better mental health among this workforce [5, 26].

This paper reports on a pilot cluster randomised controlled trial (the Wellbeing in Secondary Education (WISE) study) of an intervention that aimed to provide support for staff mental health and strengthen their ability to support students. There were two strands to the intervention: delivery of the training package Mental Health First Aid (MHFA) and the setting up of a peer support service for staff.

MHFA was devised by a team of academics and clinicians in Australia, with the aim of equipping individuals to provide help to people “in mental health crises and/or in the early stages of mental health problems” [27]. It has been evaluated in the workplace [27] and a youth version, devised for those working with teenagers, has been evaluated in high schools [28]. Several trials have demonstrated that MHFA can increase participants’ knowledge, confidence and intent to help others and reduce negative attitudes towards mental health [29], as well as directly benefitting the mental health of course participants [27]. Only three studies have measured mental health outcomes among those receiving support from trainees, none of which found positive results [30], which may be due to short follow up periods of 6 months or less. MHFA has been adapted for use in England and has become widely used. Studies have shown positive results in terms of improved knowledge, attitudes and confidence in helping [31, 32], but outcomes for potential recipients have not been measured in the English context, and no study has used a randomised design.

Moll (2014) in an in-depth study of a healthcare organisation, revealed the silence and stigma that surrounded mental health issues, and the desire of staff to have onsite support [33]. A similar culture of fear and stigma regarding asking for support has also been reported among teachers [21, 22], therefore a peer support service was introduced as an easily accessible onsite intervention for staff to access as a ‘first port of call’. It was hypothesised that this service, alongside the delivery of youth MHFA training, would not only increase the capacity to provide support to individual staff and students in need, but would create a more open school-wide culture with regard to discussion and awareness of mental health issues.

The pilot study aimed to assess the feasibility and acceptability of the intervention, and explore the justification for evaluating the intervention in a full cluster randomised controlled trial (RCT). Specifically, a mixed methods approach was taken to answer the following questions:

1. Will schools, staff and students participate in a full cluster RCT and what is the likely attrition from the intervention and the research?
2. Is MHFA training appropriate for the English secondary school context, and does it improve mental health knowledge and attitudes among school staff attendees?
3. Is a peer support service for school staff acceptable, feasible and sustainable, and what are the barriers and facilitators to it being effective?
4. What sample size is required for a full cluster RCT, with staff’s score on the Warwick Edinburgh Mental Wellbeing Scale (WEMWBS) as the primary outcome?

## Methods

### Sample

All non-fee paying, mainstream secondary schools in three adjacent local authorities (English administrative areas) were eligible for inclusion. Letters inviting expressions of interest were written to 32 head teachers and follow up phone calls attempting to contact a member of staff in charge of pastoral care and/or staff development were made. Once a sufficient number of schools - representing a range of socioeconomic catchment areas, size and academic results - had responded, no further schools were followed up.

### Design

The study was a pilot cluster randomised controlled trial with schools as the unit of randomisation. Equal numbers of schools were randomly allocated to either an intervention (*n* = 3) or control (*n* = 3). As the peer support service was available to all staff in one school, individual randomisation of teachers within schools was considered to be inappropriate.

### Random allocation and blinding

Participating schools were paired according to free school meal eligibility (FSM) as a proxy for socioeconomic catchment area – a school was coded as high FSM if the percentage of students eligible was above the national average (in 2013), and low if the percentage eligible was below this national average. The schools within each pair were randomly allocated using a computerised programme, by a statistician blinded to the actual school identities or any information about them. Allocation took place once all schools were recruited and after baseline measures were collected. Blinding of participants and the research team was not possible during the intervention, collection of process data and collection of follow up questionnaire data.

### The intervention

1. Peer support service for staff
  All staff in the intervention schools were invited to nominate colleagues who would be suitable for the role of peer supporter via self-complete questionnaires. The 8–9 staff with the most nominations - ensuring a mix of teaching/support staff, gender and seniority – who consented to take part were trained in the full two day adult MHFA course by a registered independent trainer (see www.mhfaengland.org), before setting up a confidential peer support service for colleagues. The standard MHFA course covers key facts, recognition and understanding of the most common mental disorders - depression, anxiety and psychosis – and provides attendees with a strategy for providing initial help to anyone appearing distressed or at risk of developing a mental health problem. The application of five steps known as ALGEE (Assess risk of suicide, Listen non-judgmentally, Give advice and information, Encourage professional help, Encourage self-help strategies) is a key part of this strategy that is referred to throughout the course [28]. Once the training had been completed, guidance was provided by the research team regarding the purpose of the peer support service, confidentiality, and gaining support for themselves (see Additional file 1: Appendix A), but peer support teams were encouraged to develop the detail of the service themselves according to what was most appropriate for their particular school, for example how it was advertised, and how staff accessed the help.
2. Youth MHFA training
  The full two day youth MHFA training was also delivered to up to 20 staff in each school, again by an external independent trainer. The school’s senior leadership team had control over how this opportunity was advertised, and which staff attended. The content of the course is similar to the standard course, but focuses more specifically on facts, signs and symptoms of distress and mental disorders among teenagers, making use of case studies to illustrate the particular difficulties young people may face and consider helping strategies. After receiving the training, staff returned to their usual jobs and applied the training as required in their usual interactions with students.
  Schools in the control group continued with usual practice.

### Qualitative data collection

Qualitative data were collected to explore questions of feasibility, acceptability, sustainability and perceived usefulness of both aspects of the intervention. In each intervention school, at least two training sessions (one adult MHFA and one youth MHFA) were observed, and notes taken guided by specific headings (the observations schedule), a summary of which is given in Additional file 2. In addition, focus groups, supplemented by individual interviews where groups were difficult to convene, were conducted with peer supporters, attendees of the youth MHFA training, and randomly selected teachers and non-teaching staff who had not received any training. In all schools (intervention and control), 1–2 senior leaders (those involved in supporting the setting up of the intervention in their schools) were interviewed to examine their views on participation in the study, and the ways in which staff wellbeing was addressed prior to the intervention being established in the school. The final number of observations, focus groups and interviews is shown in Table 1. The interviews with senior leaders were conducted two to four months after the MHFA training had been delivered and the peer support service set up, and the focus groups and interviews with other staff were conducted four to six months after this point.

**Table 1:** Interviews and focus groups conducted in the intervention schools

	Observations	Focus groups	Interviews
	8 adult MHFA training days 5 youth MHFA training days	5 peer supporter 3 youth MHFA trainee 3 untrained teacher 2 untrained support staff 1 untrained mixed	7 senior leaders 5 peer supporters 1 untrained teacher
Total	13 days	14 groups	13 interviews

Interviews and focus groups took place on school premises, and the length of time taken ranged from 30–50 min.

### Qualitative data analysis

All interviews and focus groups were audio-recorded and transcribed. The different groups of data (observations, interviews with key staff, interviews/focus groups with peer supporters, focus groups with attendees of youth MHFA, interviews/focus groups with non-trained staff) were initially analysed separately using constant comparison techniques common to qualitative research [34]. Initial transcripts were scrutinised for emergent themes which were compiled into a coding frame. As each new transcript underwent analysis in this way, those themes that did not fit the existing frame were either added as new themes to the coding frame, or were used to expand and modify existing themes, until all data had been accounted for. Where relevant, themes were then compared across the different groups of data for similarities and contrasts. Initial focus group and interview transcripts were analysed independently by two members of the research team, to check the reliability of the coding frame. Subsequent analyses were then conducted by one team member, with the other checking a random sample to ensure the coding frame was an accurate summary of the data.

### Questionnaire data collection

Baseline questionnaire measures were collected before schools were allocated to study arm in June-July 2013, the MHFA training was delivered and peer support services set up between September and January of 2013, and the same measures collected at follow up in June-July 2014. The questionnaires were used to examine process questions regarding learning from the training and use of the peer support service, to assess response rates to the primary and secondary outcome measures, as an indication of likely attrition in a full trial, and to gather information required to calculate sample size required for a full trial.

Staff questionnaires were completed during staff meetings or training events with a researcher present. Questionnaires for those who were not in attendance were left for individuals to complete in their own time and return to the study team. Student questionnaires were completed in class, or in larger groups in a school hall. Students were in years 8 and 9 at baseline (12–14 year olds), and years 9 and 10 at follow up (13–15 year olds). These year groups were selected as the minimum age in which the wellbeing outcome measure has been validated [35], and to avoid years in which exams were prominent.

### Staff questionnaire measures

The following measures were taken at baseline and follow up.

Staff wellbeing was measured using the Warwick Edinburgh Wellbeing Scale (WEMWBS) [6]; a 14 item scale measuring both subjective and psychological wellbeing, short enough to be used in population level surveys, responsive to change and validated among community samples of adults in the UK. A higher total score means greater wellbeing (maximum score = 70). This was the primary outcome, and therefore was used to calculate the required sample size in a full trial.

Staff depression was measured using the PHQ-9; a scale that is suitable for measuring levels of depressive symptoms in population-based studies and is short enough to be used in self-complete surveys. Individuals received an overall score, in which the higher the score the more severe the depression (maximum score = 27). Responses were also categorised as depressed or not depressed, using a cut off of 10 or more [7]. This was a secondary outcome.

Learning from the MHFA training was examined using evaluation tools developed by the founders of MHFA and used in previous evaluations [27, 28]. Knowledge was tested via 12 statements about mental health, with the option to select true/false/don’t know. Examples were “it is not a good idea to ask someone if they are suicidal in case you put the idea in their head” and “it is best to get someone having a panic attack to slow down their breathing”. A point was given for each correct answer, with “don’t know” or the wrong answer scored as 0. Stigmatising attitudes were examined using ten statements relating to young people in two vignettes, one suggesting symptoms of depression, the other symptoms of anxiety (Additional file 3: Appendix B). Examples were “a problem like Emma’s is a sign of personal weakness” and “people with a problem like Paul’s are unable to contribute much”. Response options were strongly agree/agree/neither agree nor disagree/disagree/strongly disagree, scored 0, 1, 2, 3, 4 respectively, with a higher score representing a less stigmatising attitude. Application of ALGEE (as described above) was measured by asking respondents to describe narratively what they would do to help the individuals in the vignettes. Responses were scored according to each step of ALGEE, with up to two points possible for each step, giving a maximum of 10 points. In the interests of keeping the questionnaire a reasonable length, separate vignettes involving adults were not included; the young people vignettes were considered suitable for measuring learning from the adult as well as the youth MHFA. Finally, questions were asked about confidence in and actual helping behaviour towards colleagues and students over the past academic year.

In addition, a series of questions were asked of the intervention group only at follow up about the peer support service: i) whether they would use it if in need ii) whether they had used it iii) why they had not used it if applicable iv) whether it was helpful if applicable.

### Student questionnaire measures

Student wellbeing was measured using the WEMWBS.

Student mental health difficulties were measured using the self-report Strengths and Difficulties Questionnaire (SDQ) [36]. A total difficulties score was calculated (maximum = 40), in which the higher the score the greater the difficulties.

In addition, all respondents were asked their gender, ethinicity, whether they receive free school meals (students only), and their role and years’ of experience in school (teachers only). Asking these questions enabled us to examine understanding and response rates – in a full trial interactions may be examined between these demographic variables and any intervention effects.

### Statistical analysis

All analyses took account of clustering by school using robust standard errors. Analyses were conducted using Stata version 14.

#### Impact of the MHFA training

Knowledge and attitudes towards mental health, application of ALGEE as well as confidence and frequency in helping a colleague and helping a student, and wellbeing/depression were all examined using linear or logistic regression models, adjusted for the appropriate baseline measure. In each case, the variable at follow up (those listed above) was compared between those who had received the training and the rest of the staff sample.

#### Impact and use of the peer support service

Use of the peer support service, as reported in the follow up intervention schools questionnaire, was examined. Narrative reasons given in the questionnaire for non-use of the service were coded according to emergent categories, and the number of times each response was given was counted.

Although we collected outcome data from participants, this was for the purposes of checking the feasibility of using these measures, and to calculate sample size needed for a full trial. As a pilot, the study was not powered to measure effectiveness of the intervention, therefore we do not report the results of the outcomes at follow up in the main body of this paper. A brief summary is available in the Additional file 4.

## Results

The results are reported under four headings, relating to the four research questions.

### Will schools, staff and students participate in a full cluster RCT and what is the likely attrition from the intervention and the research?

**Fig. 1:**
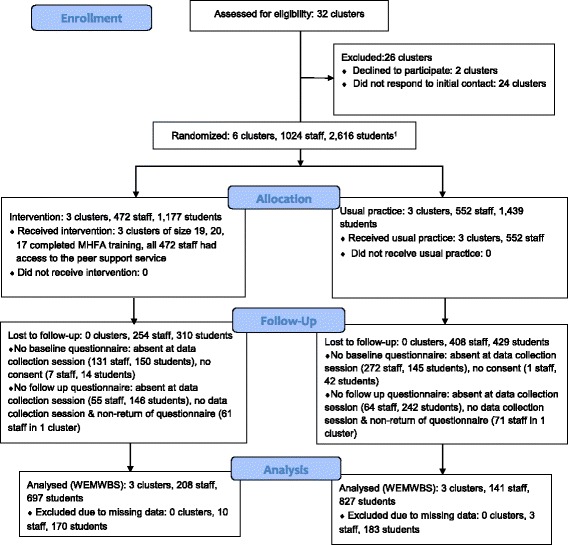
Flow diagram of participants through the study

Figure 1 shows the flow of schools and individuals throughout the study. Of the 32 eligible schools, 8 responded to the initial letter or follow up phone call, of whom 2 subsequently choose not to proceed. The remaining 6, containing a total of 1024 staff and 2,616 students in years 8 and 9, consented to participation.

All 6 schools remained in the study, and received the allocated intervention. In the intervention schools, all 56 staff who agreed to attend the MHFA training completed the course. The staff selected and trained to be peer supporters (*n* = 25) remained so until the end of the study. All staff in the intervention schools had access to the peer support service from the time of set up to the study end.

At baseline, 613 (59.9 %) staff completed the questionnaire (74.1 % teaching staff and 40.2 % support staff). Of those, 362 completed the follow up questionnaire and 349 had complete data for the main outcome the WEMWBS. A further 286 staff who had not completed a baseline questionnaire completed the follow up one, making a total of 648 staff with at least some follow up data.

In total, 2265 (86.6 %) students completed the baseline questionnaire. Of those, 1,877 completed the questionnaire at follow up and 1,524 had complete data for the WEMWBS outcome. A further 222 students who had not completed a baseline questionnaire completed the follow up one, making a total of 2,487 students with at least some follow up data.

There was greater attrition among staff in the control schools compared to staff in the intervention schools (74.5 % versus 55.9 %). This was not the case among students, where attrition was similar across arms (42.5 % in control compared to 40.8 % in intervention).

There was no difference in baseline WEMWBS and PHQ-9 scores among staff who had follow up data and those who did not. Among students, those who had data at both time points had slightly higher wellbeing than those who did not (mean = 47.8 [95 % CIs: 47.4, 48.2] versus mean = 46.3 [95 % CIs: 45.4, 47.1]), and a slightly lower total difficulties score (mean = 11.7 [95 % CIs: 11.3, 12.0] versus mean = 12.8 [95 % CIs: 12.2, 13.4].

### Is MHFA training appropriate for the English secondary school context, and does it improve mental health knowledge and attitudes among school staff attendees?

#### Qualitative findings

Both the adult and youth versions of the MHFA training were reported to be useful for conferring new knowledge and skills, giving reassurance about current practice, providing opportunity to discuss difficulties in supporting students with colleagues and developing awareness of one’s own mental health:

“the way I listen I think is a bit different, because of the training you suddenly think oh there’s something, she’s not just talking to me about how her husband broke her favourite plate it’s something below, there’s something else there”

*Adult MHFA attendee*

“For me it was that reassurance, I thought OK it’s what I normally do, I think I’m on the right track”

*Youth MHFA attendee*

Those who were offered the opportunity by their schools to attend the youth MHFA course during the pilot were a mixture of teaching staff but also more specialist support staff such as learning mentors. Several attendees suggested that such specialist support staff, and also experienced teaching staff, already had much of the knowledge and skills covered, but that less experienced teaching staff who were often the first to encounter students in difficulty in their role as tutors would benefit a great deal from the course:

“I think it’s useful, within my year team there are people who I think are less confident and I’ve seen a difference in their practices”

*Youth MHFA attendee*

There was a feeling among participants that student mental health is deteriorating, with pressures about exams, times of transition, friendship issues, parental conflict and self-image being listed, and youth MHFA was seen to be very relevant in that context. There was also a strong sense among course attendees, but also among senior leaders and non-trained participants, that teachers are under a great deal of pressure - the main sources of which were cited as student performance (as a reflection of their own performance), inspections by the government’s Office for Standards in Education (Ofsted), balancing work and home life, and supporting vulnerable students – and that supporting their mental health is important:

“I believe that maintaining mental wellbeing is as important as maintaining physical wellbeing for ability to work and ability to function really well. Because as a teacher, you have to be functioning at your best at all times when you’re in front of a class, and there’s no, there’s no leeway for that. Even more so now, because you’re expected to, you know, create all these outstanding lessons, engagement with young people is supposed to be, you know, A1”

*Senior leader*

One suggestion for improvement to the youth MHFA training was making the course shorter to ensure it was accessible to all teachers; it was suggested that many schools may be reluctant to release mainstream teachers for two days’ training, and pay the cover required. A second suggestion was to reduce some of the focus on facts and spend more time considering skills and strategies for providing support.

#### Questionnaire results

Of the follow up questionnaire respondents, 22 had completed the adult MHFA course, 24 had completed the youth MHFA course, and 4 had completed both, making a total of 46 respondents who had completed at least one of the courses (82.1 % of all those who completed the training). 15 individuals answered “can’t remember” as to whether they had attended either course and were omitted from the analysis. As the questionnaires were anonymised it is unclear whether any of these 15 had attended any MHFA training as part of the WISE study.

Table 2 shows mental health knowledge and attitudes at baseline and follow up. At baseline, those who went on to attend the training already had slightly better knowledge, less stigmatising attitudes towards those with depression and anxiety and a greater tendency to use ALGEE compared with the rest of the staff. At follow up, once baseline scores were adjusted for, those who had received the training had better knowledge about and less stigmatizing attitudes towards mental health difficulties compared to the rest of the staff in all schools (*p* values range from <0.01–0.04). Trained staff were also more likely to apply ALGEE to both vignettes at follow up once baseline scores were adjusted for, compared to untrained staff in all schools (difference = 0.81[0.13, 1.49], *p* = 0.03 and 0.57 [0.02, 1.17], *p* = 0.06). Table 2 also shows confidence and reported helping behaviour towards colleagues and students. At baseline, those who went on to receive the training already had higher confidence in helping colleagues and students and reported more frequent helping behaviour compared to those who did not receive the training. At follow up, this group were more likely to have high confidence in helping a colleague (OR = 6.41 [3.00, 13.70], *p* < 0.01), although there was no difference in actual helping behaviour reported in the past academic year.

**Table 2:** Mental health knowledge, attitudes, confidence in helping and self-reported helping behaviour at follow up comparing those who had completed MHFA training with the rest of the sample^a^

	Mean score at baseline (SD)	Mean score at follow up (SD)	Difference at follow up (95 % CIs)^b^
Knowledge Statements^c^			
Whole sample no training (*n* = 175)	6.5 (1.9)	6.6 (2.0)	1.84 (0.13, 3.56)
Staff who completed training (*n* = 19)	7.3 (1.9)	8.9 (2.0)	*p* = 0.04
Attitudes – Depression Vignette^d^			
Whole sample no training (*n* = 280)	32.2 (4.3)	32.3 (4.6)	1.87 (0.93, 2.80)
Staff who completed training (*n* = 36)	34.2 (3.8)	35.5 (3.6)	*p* < 0.01
Attitudes – Anxiety Vignette^d^			
Whole sample no training (*n* = 286)	33.1 (4.8)	33.4 (4.5)	2.01 (0.42, 3.59)
Staff who completed training (*n* = 35)	34.9 (3.9)	36.4 (3.7)	*p* = 0.02
Application of Algee – Depression Vignette^e^			
Whole sample no training (*n* = 284)	3.0 (1.5)	2.9 (1.6)	0.81 (0.13, 1.49)
Staff who completed training (*n* = 35)	3.5 (1.7)	3.9 (1.7)	*p* = 0.03
Application Of Algee – Anxiety Vignette^e^			
Whole sample no training (*n* = 267)	2.5 (1.4)	2.5 (1.4)	0.57 (−0.02, 1.17)
Staff who completed training (*n* = 34)	2.7 (1.5)	3.2 (1.6)	*p* = 0.06
	*N* (%) at baseline	*N* (%) at follow up	OR at follow up (95 % CIs)^b^
Moderate - High Confidence Helping a Colleague^f^			
Whole sample no training (*n* = 179)	179 (57.2)	172 (55.0)	6.41 (3.00, 13.70)
Staff who completed training (*n* = 38)	29 (76.3)	34 (89.5)	*p* < 0.01
Moderate - High Confidence Helping a Student^f^			
Whole sample no training (*n* = 312)	175 (56.1)	188 (60.-3)	4.23 (0.88, 20.32)
Staff who completed training (*n* = 38)	35 (92.1)	35 (92.1)	*p* = 0.07
Helped a Colleague at Least Once a Month in the Past Academic Year^g^			
Whole sample no training (*n* = 149)	149 (48.4)	142 (46.1)	1.26 (0.86, 1.85)
Staff who completed training (*n* = 36)	27 (75.0)	23 (63.9)	*p* < 0.23
Helped a Student at Least Once a Month in the Past Academic Year^g^			
Whole sample no training (*n* = 310)	185 (59.7)	181 (58.4)	2.35 (1.72, 3.22)
Staff who completed training (*n* = 37)	34 (91.9)	32 (86.5)	*p* < 0.01

^a^All analyses include only the subsample who have data at both time points

^b^Adjusted for baseline measure

^c^Maximum possible score of 12

^d^Maximum possible score of 40

^e^Maximum possible score of 10

^f^Response to question ‘How confident would you feel in helping a colleague/student who appears stressed or down?’ High (moderately, quite a bit, extremely) versus low (not at all, a little bit)

^g^Response to question ‘In the past academic year, how often have you provided emotional support to a distressed colleague/student?’ Once a month or more versus less than once a month

### Is a peer support service for school staff acceptable, feasible and sustainable, and what are the barriers and facilitators to it being effective?

#### Questionnaire results

In total, 19 respondents (6.3 % of those who completed the follow up questionnaire in the intervention schools) had used the peer support service, with 17.4 % saying they would use it in response to the question “If a work related problem was making you stressed or down, who would you talk to about it at school?” Of those who did use the service, 14 (73.7 %) found it helped and 5 (26.3 %) found it helped a lot.

Table 3 shows reasons given by respondents for not using the service. The commonest reason was that help was not needed, followed by a preference for talking to other colleagues and lack of knowledge about the service. Other less common reasons included concerns about confidentiality, not wanting to approach those particular staff, and choosing to access support from outside school.

**Table 3:** Reasons given for not using the peer support service among intervention school respondents

Reason	Responses^a^ *N* (%)
Did not need to	96 (32.1)
Prefer to talk to other colleagues/work friends	59 (19.7)
Lack of knowledge about it	56 (18.7)
Wouldn’t approach those particular people	16 (5.4)
Concerns about confidentiality	14 (4.7)
Didn’t think it would help/they are not professionals	13 (4.3)
Not enough time at work/chosen supporter not available	12 (4.0)
Access support outside of school	10 (3.3)
Reluctant to discuss issues at work, concerns about being judged	8 (2.7)
Felt uncomfortable approaching them	5 (1.7)
Other	10 (3.3)
TOTAL REASONS GIVEN	299

^a^Where individuals gave more than one reason, all reasons were counted separately

#### Qualitative results

Peer supporters discussed a range of ways in which the service had supported staff, from providing a sounding board, through early intervention to prevent escalation of a problem, to organising professional help for individuals in a great deal of distress:

“Often people just really do need somebody to listen to them and spend a little bit of time and care over what’s going on for them. You don’t necessarily need a resolution”

“I suggested to her to see a GP, and it’s a long-term sort of process of recovery but we had a long long chat on the phone and she could not cope anymore, she said “I cannot be in school anymore””

In all the schools, the peer support service tended to be used in an immediate and informal manner, rather than through official, planned appointments:

“people do just say informally in the corridor have you got 5 min can we have a chat and you sort of work out whether it’s dire and they need that chat now, or you sort of say well could you come in half an hour and I can give you some time”

This approach appeared to suit the non-stop and unpredictable nature of school life, in which staff often did not even share the same lunchbreaks, and rarely had much time away from their duties.

Both peer supporters and senior leaders indicated that the existence of the service had raised general awareness about mental health issues in their schools, thus encouraging more open discussion, and reassuring staff that their wellbeing mattered to their employer:

“I think it sends a really big message out to staff in general, they’re seeing posters saying a message which is we care about you, there is a network there for you if you need it”

*Senior leader*

There was some discussion about the impact of being a peer supporter, both positive, in terms of the affirming feelings generated by “making a difference”, and negative, due to the upset experienced when listening to what colleagues had to say, and the additional demands on individuals’ time. However, these concerns may have been mitigated by the fact that the peer supporters tended to already be those to whom others went for support, so becoming a peer supporter may to some extent have formalised pre-existing support networks, rather than created new ones:

“There are people that you move towards who radiate support and then you’ve got the people who if you sit down next to them, it’s like you get it all sucked out of you, so there’s radiators and drains, and if I look at the members of staff who have been nominated for this, they’re all the radiators”

Among the focus groups of untrained staff, three people shared personal experience of having used their peer support service; one discussed the value of having someone to talk to outside their usual work relationships where they felt they always needed to “exude calm”, another appreciated having the support mechanism as a new teacher, and the third described the relief that contact with a peer supporter provided at a time of emotional crisis:

“Yeah it was them effectively giving me a big hug, and protecting me from it until I was ready to go back in the classroom, and I was fine, and I’ve been fine from that. But if I hadn’t have had that intervention, if I’d had to go in the class and sort of stifle all those feelings in front of the children, you know, then it would have been a different situation probably”

However, barriers to using the service emerged from discussions which partially echoed those identified in the questionnaires, in particular a lack of awareness about it, not wanting to discuss problems at work due to concerns about confidentiality or being judged, a preference to go to pre-existing support networks in school, and a concern that they would be a burden on the peer supporter when everyone was working under such pressure. What became clear was that the likelihood of the service being used by an individual depended on the nature of the problem, the right combination of people as supporters, and the extent to which they had other places to turn. Time for the service to become tried and tested was also raised as an important factor by potential users:

“I think you know, it’s maybe human nature to be a little suspicious of something new to start with, and you know, the kind of reactions like “well I’m not gonna speak to anybody” might change when you’re in a situation that you do need to speak to somebody, so I think you know, it’s still in its very early stages and it’s almost like people have got to have used it, say it was really good, you should go”

A number of suggestions arose for improving the peer support service: ensuring the peer supporters were adequately supported, developing a strategy for regularly promoting the service to all staff, and identifying a member of the senior leadership team to ‘champion’ the service, to help ensure sustainability:

“I think someone on the senior leadership team needs to be involved in the project not as a staff supporter because I think our school is like others that would immediately create a barrier to any sort of free chat or anything, but to oversee it to make sure it is implemented and happens”

### What sample size is required for a full cluster RCT, with staff’s score on the Warwick Edinburgh Mental Wellbeing Scale (WEMWBS) as the primary outcome?

Table 4 shows key information for the primary outcome staff WEMWBS score. The mean baseline WEMWBS score by school ranged from 46.8–49.4 for all staff, and 46.0–49.5 among teachers only. The ICC was 0.01 (95 % CIs 0.00, 0.03) among all staff, and 0.01 (95 % CIs 0.00, 0.05) among teachers only, indicating relatively low levels of clustering. Assuming the WEMWBS scores and numbers of respondents reported here, a sample size of 24 schools (12 intervention and 12 control) would achieve more than 90 % power at the 5 % significance level to detect a difference in WEMWBS score of 3 points if the ICC was 0.01, and 80 % power to detect such a change if the ICC was 0.05, which is the highest upper 95 % CI limit of the ICCs found here. A change of 3 points in the WEMWBS is the minimum meaningful change discussed in a WEMWBS user guide [37]. It is also close to the difference in mean scores between the lowest and the highest ranked schools in this pilot study.

**Table 4:** Key information for the primary outcome staff WEMWBS score

	Cluster size (mean no. with data at both time points)	Mean baseline WEMWBS score (SD)	ICC for baseline WEMWBS score (95 % CIs)
All staff	73	48.5 (8.3)	0.01 (0.00, 0.03)
Teachers only	55	48.0 (8.4)	0.01 (0.00, 0.05)

## Discussion

### Main findings

The results show that it is possible to recruit schools to a study that focuses on secondary school staff’s own mental health and wellbeing, and their skills in supporting vulnerable students. Although only a quarter of eligible schools responded to the initial contact, we did not actively follow-up non-responders as we only needed to recruit 6 schools to the pilot. A future randomised controlled trial would need to explore alternative ways to engage with relevant school staff, for example through attending local schools network meetings focusing on relevant topics such as school health or inclusion. A sample size calculation, based on the information collected in this pilot study, indicated that a definitive trial would require a sample of 24 schools to have sufficient power to detect a 3 point difference in teacher WEMWBS scores.

Few participants refused to complete a questionnaire – or in the case of students had parents who opted out. However, numbers of staff and students with data at both time points were below 50 %, partly due to staff in particular not being present at the data collection session, and also due to individuals leaving schools before follow up. To address the former issue, time and costs should be built in to a future trial to enable a number of return data collection visits at each time point. Analyses that do not require data at all time points should be considered to address the issue of individuals leaving, for example cross sectional analyses of successive cohorts of students.

Response rates among staff were poorer in the control arm. This was partly because the largest school in the study, which happened to be in the control arm, was unable to arrange a data collection session during directed time (when staff were required to attend) at both data collections. Further, a second control school arranged data collections during meetings where only teaching staff were required to attend, so response rates among non-teaching staff were particularly low. It is possible that control schools were less engaged in the study than intervention schools; however the fact that student response rates were comparable does not support this.

Qualitative and quantitative findings indicate that both the adult and youth MHFA courses were considered relevant to the mental health of both staff and students, and were effective at improving knowledge, attitudes, confidence and skills in supporting others. It is important to note that in the analyses of the questionnaire data, those who received the training were a non-random sample – they already scored more highly at baseline for knowledge, non-stigmatising attitudes, confidence in helping and actual helping behaviour than those who did not go on to receive the training. Nevertheless, the trained group did show greater improvements in most of these things following the training, compared to the untrained staff, in keeping with previous studies that have shown such effects for MHFA training [29]. The fact that trainees had higher confidence in supporting students and were already more likely to be doing this at baseline relates to the qualitative finding that the youth MHFA course was potentially difficult for non-specialist teachers to access due to its length. A future trial would need to ensure that the training targets those who would benefit the most; for example tutors or year heads are likely to have the most regular contact with students by virtue of this role, and yet are unlikely to have received training in supporting those in mental health difficulty [22]. Reducing the length of the youth MHFA training would make it more accessible for mainstream teachers, as schools would find it easier to release teachers and pay for cover for one day rather than two. There is a trade-off between keeping the training a reasonable length and ensuring it is sufficiently in-depth, but if the reduced length training can also be more ‘streamlined’ to suit the needs of teachers (e.g. focusing more on skills and strategies relevant to the school context) it is likely to remain of value to teachers in the limited time they have available for training.

It is also of note that those who went on to receive training reported relatively high levels of helping a colleague at baseline, suggesting that the system of staff nominating colleagues to become peer supporters identified those who were already fulfilling this role informally. This means the intervention may have equipped those already providing help to do this more effectively – particularly as this group’s confidence in helping colleagues rose substantially at follow up - rather than greatly increasing the workload of those selected to become peer supporters. The qualitative data provides further evidence that peer supporters were people already likely to be providing support to colleagues.

The staff peer support service was generally viewed by senior leaders, peer supporters, and potential and actual recipients as making a positive difference both at the individual level for those who sought support, and at a whole school level through the positive messages that its existence conveyed. However, a number of barriers to using the service and suggestions for improvement were identified, that would need to be addressed in any future intervention. More regular advertising of the service and increasing the proportion of staff acting as peer supporters would increase the likelihood that all staff would know about the service and be comfortable approaching at least one supporter, and may mitigate concerns about being a burden on a few busy colleagues. Although the evidence indicated that the peer supporters already tended to provide a lot of support informally, ensuring that they feel adequately supported was clearly a concern that would need to be addressed in a future study, for example through the involvement of local public health or mental health professionals already working with schools. Sustainability of the peer support services was not examined in this study due to the short follow up time, but ensuring Senior Leaders visibly support the service, re-launching it every academic year and finding ways for it to become embedded within school systems were all suggestions arising from the qualitative evaluation that are likely to help ensure this.

### Study limitations

A limitation of the study was that less than 50 % of staff and students had questionnaire data at both times points; as noted above this could be addressed in a future study by conducting a statistical analysis that does not require a complete dataset. For example, provided intervention and control arms are sufficiently balanced at baseline, an analysis of mean scores at follow up, without controlling for baseline, could be conducted. Relatedly, the control arm had a particularly low staff response rate compared to the intervention arm. As noted above, this was due to two of the three control schools failing to provide directed time for all staff to complete the questionnaires. In a larger trial, one or two schools would not have such an influence on response rates. All participating schools struggled to some extent to gain a good response rate from non teaching staff as they were less likely to attend meetings. An option for a future trial would be to focus only on teaching staff, given that this is the group found to be at risk of poor emotional health and wellbeing [5]. A second limitation is that the comparison between those who attended the training and those who did not involved non-random samples. This could be addressed in a future study by using a Complier Average Causal Effect (CACE) approach (using Instrumental Variable analysis or Principal Stratification) [38] to examine the impact of MHFA training among those who would have completed the MHFA training in the control schools had they been offered it, and compare this to the actual impact among course attendees. Thirdly, the follow up data were collected approximately six months after the training was delivered and the peer support service had been set up, which, according to the qualitative evidence, was not long enough for the peer support service to become embedded within school life and therefore well used.

### Relevance to the wider literature and implications

A number of school-based randomised controlled trials have evaluated mental health interventions, but these have generally focused on improving student outcomes only, and have produced limited evidence of effectiveness [39, 40]. A small number of studies have aimed to address teacher training needs in supporting students. The Incredible Years Teacher Classroom Management programme focuses on increasing teachers’ skills in supportive classroom techniques, and pilot trials in the UK have reported promising results in terms of reduced negative behaviours among children with poorer mental health, a reduction in teachers’ negative behaviours towards such children, improved teacher self-efficacy and improved teacher emotional health [41, 42]. However, this programme is currently limited to primary school-aged children. The Seyle study [43], a randomised controlled trial across ten European countries in secondary schools, compared the effectiveness of training teachers to recognise and support students at risk of suicide, with raising student awareness about mental health and suicide and screening by professionals, but found that only the student training intervention had an impact on suicide ideation and attempts. As noted in the introduction, one previous trial has evaluated the impact of delivering youth MHFA into schools, and found positive changes in staff mental health knowledge, attitudes and confidence in helping, but no change in reported helping behaviours or student mental health [28]. However, neither of these trials directly supported the mental health of the teachers themselves. The authors of the Seyle study found that teachers with poor wellbeing were less likely to believe they could help students with emotional or behavioural problems, and they argue that this may have explained the ineffectiveness of the teacher training intervention [44].

## Conclusions

**Fig. 2:**
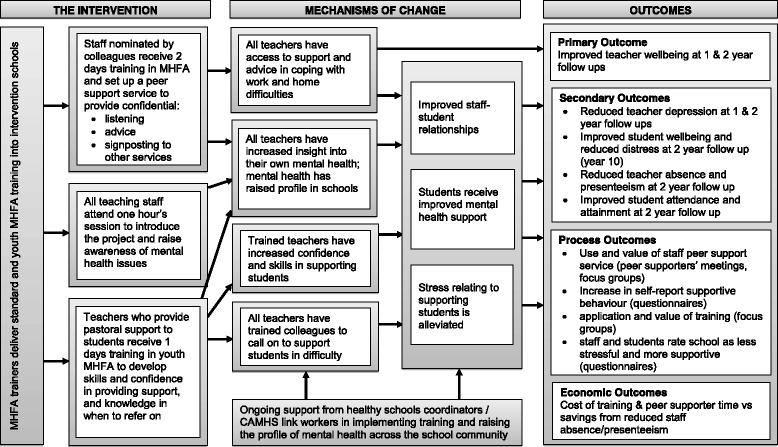
Logic Model of the WISE intervention

Our study builds on a body of work focused on improving mental health within schools, but is unique in delivering mental health support as well as training to secondary school teachers. Given the findings - that schools can be recruited for such a trial, and that both the MHFA training and the staff peer support elements of the intervention are feasible, and are perceived by school staff to be acceptable and relevant to this context - the next step, following the MRC guidance for the evaluation of complex interventions [45], will be developing a full cluster RCT of the WISE intervention, sufficiently powered to evaluate the impact on staff mental health and wellbeing. A logic model setting out the intervention, theorised mechanisms of change and outcomes on which this full trial would be based is included in Fig. 2. The trial will include an integral process evaluation, to enable understanding of whether changes in the primary and secondary outcomes were due to the mechanisms of change and more proximal outcomes specified in the logic model.

The pilot has highlighted the areas in which such a cluster RCT may encounter challenges, but also, where it worked well, we were able to identify best practice to avoid those challenges. To ensure enough schools can be recruited, we will identify a senior leader in the school who oversees staff wellbeing and/or continuing professional development; we will work with schools from the start regarding expectations of response rates and to identify meeting times in which to collect data and appropriate strategies for following up absentees; we will adapt the training offered as part of the intervention to ensure it is more accessible to mainstream teachers and work with schools at the recruitment stage to identify in-service training time during which it can be delivered; and we will work with senior leaders in the intervention schools to ensure the peer support service is appropriately championed and advertised among staff. With these improvements in place from the outset, a cluster RCT will be the most appropriate way to examine if this intervention has an impact on teacher and student wellbeing and mental health.

## Additional files

Additional file 1: Guidelines for Peer Supporters. (DOCX 15 kb)
Additional file 2: Summary of topics for qualitative data collection. (DOCX 17 kb)
Additional file 3: Vignettes. (DOCX 20 kb)
Additional file 4: Results of the main outcomes at follow up. (DOCX 71 kb)

## Abbreviations

CACE: Complier Average Causal Effect
CI: Confidence Intervals
FSM: Free School Meals
GP: General Practitioner
ICC: Intra Cluster Coefficient
MHFA: Mental Health First Aid
MRC: Medical Research Council
Ofsted: Office for Standards in Education
PHQ-9: Patient Health Questionnaire – 9 item
RCT: Randomised Controlled Trial
SDQ: Strengths and Difficulties Questionnaire
WEMWBS: Warwick Edinburgh Mental Wellbeing Scale
WISE: Wellbeing in Secondary Education
