# Supplementary material for: A pilot cluster randomised controlled trial of a support and training intervention to improve the mental health of secondary school teachers and students – the WISE (Wellbeing in Secondary Education) study
Source: BMC Public Health. 2016 Oct 6;16:1060. doi: 10.1186/s12889-016-3737-y (PMC5053067; doi:10.1186/s12889-016-3737-y)
Supplement: Additional file 1: — Guidelines for Peer Supporters. (DOCX 15 kb) [file 12889_2016_3737_MOESM1_ESM.docx]

| The WISE Project 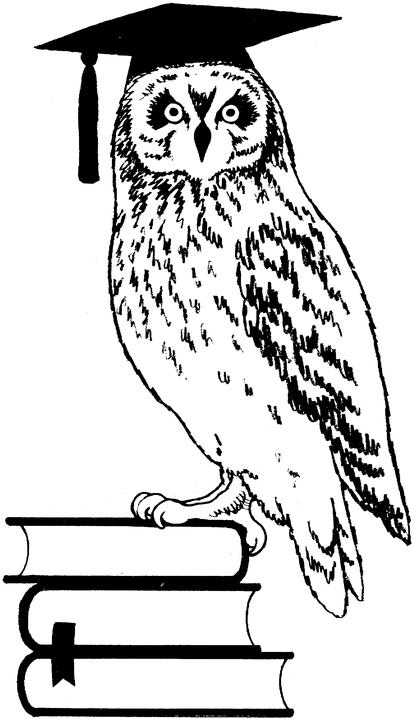 Wellbeing In Secondary Education |  |
| --- | --- |

# **Guidelines for Peer Supporters**

Thank you very much for agreeing to be a peer supporter for your school. The purpose of your role is to form part of a team providing a confidential one-to-one support service to colleagues, offering a listening ear, and, where appropriate, signposting to other services.

Below is a series of suggested guidelines for peer supporters, which we hope will help the service to run as smoothly and effectively as possible.

- We advise you to have a ‘confidentiality policy’ amongst your team, which states that confidentiality of all staff members who approach the service for help will be respected, even between team members. You should include in this policy any exceptional circumstances in which confidentiality may need to be broken, for example if there is a concern that a student may be at risk, and what your procedure should be in this event. You should base such decisions on usual school policy and procedures – we do not expect you to keep things confidential where school policy would dictate otherwise. However, we would expect that in the vast majority of cases, confidentiality can and should be maintained.
- We encourage you to have regular team meetings, to swap ideas and to discuss any problems or concerns arising from a one-to-one session, or about being a peer supporter in general. **Confidentiality of individuals you have supported should be maintained during such discussion.** For this to happen, we suggest that you elect a team member to coordinate these meetings.
- We encourage you to organise yourselves into “buddy pairs”. Each pair would provide ongoing informal support and advice to each other as and when required. Again, **confidentiality of individuals you have supported should be maintained during any discussion between buddies.**
- We suggest that you allow staff members to choose who they would like to approach for a one-to-one peer support session.
- You will need to decide how to advertise the service within the school community. We will be providing leaflets and posters to help with this. You may also want to present in team meetings etc. The information you provide should make your confidentiality procedure clear.
- Please remember to respond to the emails from the study team, asking for data about who you have helped over a set period. Please include any time where you have offered some support, even if this was just informally on a corridor. We will not be asking for personal data about the colleagues you have helped.

Please get in touch with a member of The WISE Project team if you have any questions or concerns about our research project.
