# Supplementary material for: A pilot cluster randomised controlled trial of a support and training intervention to improve the mental health of secondary school teachers and students – the WISE (Wellbeing in Secondary Education) study
Source: BMC Public Health. 2016 Oct 6;16:1060. doi: 10.1186/s12889-016-3737-y (PMC5053067; doi:10.1186/s12889-016-3737-y)
Supplement: Additional file 2: — Summary of topics for qualitative data collection. (DOCX 17 kb) [file 12889_2016_3737_MOESM2_ESM.docx]

Web Box 1: summary of topics explored through observations, focus groups and interviews

| **Observations**   - Physical setting of the training session e.g. how comfortable, how seating was arranged - Any practical difficulties that arose in the smooth running of the session e.g. any participants having to leave early - Signs that participants were or were not engaged e.g. body language, participation in discussion - Questions / topics raised and how well these were addressed by the trainer / course content - Extent to which issues relating to schools were covered and how much participants drew on their experience at work - Extent to which participants appeared to have formed as a team by the end |
| --- |
| **Senior contact interviews**   - How far there is a need to focus on staff wellbeing - How staff are supported in this school – gain specific examples of activities, services - Barriers and facilitators to supporting staff prior to the intervention and ongoing - Mental health training available to staff in this school - Experience of participating in this study |
| **Peer supporters focus groups / interviews**   - Experience of being nominated and taking part - Support they were already providing and what has or has not changed - Reflections on the MHFA; practical aspects, content, value, ideas for improvement - How learning from the course has been applied - The kind of help provided as a peer supporter - Practicalities of the service e.g. advertising, how they are contacted, where they speak to people - Barriers / facilitators to the service continuing - Support they receive - Any wider changes in the school’s culture regarding support for staff |
| **Youth MHFA attendees focus groups / interviews**   - How they came to attend - Reflections on youth MHFA; practical aspects, content, value, ideas for improvement - Support they provide to students and how learning from the course has been applied - Whether they have shared learning with colleagues - Reflections on the peer support service: practicalities, perceived value, would they use it? - Any wider changes in the school’s culture regarding support for staff - Reflections on completing the WISE study staff questionnaire |
| **Untrained staff focus groups / interviews**   - Why they did not attend the training, their views on the training – do they perceive that it would have been useful for them? - Have they learnt anything from colleagues who did attend - Support they provide to students - Reflections on the peer support service: practicalities, perceived value, would they use it? - Any wider changes in the school’s culture regarding support for staff - Reflections on completing the WISE study staff questionnaire |
