# Supplementary material for: A pilot cluster randomised controlled trial of a support and training intervention to improve the mental health of secondary school teachers and students – the WISE (Wellbeing in Secondary Education) study
Source: BMC Public Health. 2016 Oct 6;16:1060. doi: 10.1186/s12889-016-3737-y (PMC5053067; doi:10.1186/s12889-016-3737-y)
Supplement: Additional file 3: — Vignettes. (DOCX 20 kb) [file 12889_2016_3737_MOESM3_ESM.docx]

Vignette 1

Emma is a 15 year old who has been feeling unusually sad and miserable for the last few weeks. She is tired all the time and has trouble sleeping at night. Emma doesn’t feel like eating and has lost weight. She can’t keep her mind on her studies and her marks have dropped. She puts off making any decisions and even day-to-day tasks seem too much for her. Her parents and friends are very concerned about her.

Vignette 2

Paul is a 15 year old living at home with his parents. Since starting his new school last year he has become even more shy than usual and has made only one friend. Although Paul’s work is OK he rarely says a word in class and becomes incredibly nervous, trembles, blushes and seems like he might vomit if he has to answer a question or speak in front of the class. At home, Paul is quite talkative with his family, but he never answers the phone and he refuses to attend social gatherings. He knows his fears are unreasonable but he can’t seem to control them and this really upsets him.
