# Supplementary material for: A pilot cluster randomised controlled trial of a support and training intervention to improve the mental health of secondary school teachers and students – the WISE (Wellbeing in Secondary Education) study
Source: BMC Public Health. 2016 Oct 6;16:1060. doi: 10.1186/s12889-016-3737-y (PMC5053067; doi:10.1186/s12889-016-3737-y)
Supplement: Additional file 4: — Results of the main outcomes at follow up. (DOCX 71 kb) [file 12889_2016_3737_MOESM4_ESM.docx]

**Results of the main outcomes at follow up**

Statistical Analyses

Analyses were carried out in Stata version 13, and took account of clustering by school using robust standard errors.

For the staff outcomes, Linear regression models were performed to examine the mean outcome score (WEMWBS/PHQ) at follow up by arm, adjusted for baseline outcome score and school FSM, as the sample were originally stratified and then paired according to this variable [1]. The models were run including the whole sample, and again including teachers only (i.e. excluding support and administrative staff).

For the student outcomes, linear regression models were performed examining the mean outcome score (WEMWBS / SDQ total difficulties) at follow up by arm, adjusted for baseline outcome score and FSM. The models were also adjusted for school year as age is likely to be associated with outcome and a missing data analysis revealed differences between arms in terms of year groups [2].

Results

Table 1 shows the results for staff outcomes. Once baseline scores and school level FSM were taken into account, there were no large differences between study arm for wellbeing or depression scores in the whole staff sample, or among teachers only, although there were small trends favouring the intervention group.

Table 1: comparison of mean wellbeing and PHQ-9 scores for staff at follow up in intervention and control arms, adjusted for baseline score and FSM

|  | | Wellbeing (n=349 all staff, 267 teachers only) | | Depression (n=319 all staff, 243 teachers only) | |
| --- | --- | --- | --- | --- | --- |
|  | | Mean WEMWBS score (SD) | Difference  (95% CIs)^1^ | Mean PHQ-9 score (SD) | Difference  (95% CIs)^2^ |
| All staff (n=349) | Control | 47.2 (8.9) | 0.35 (-2.03, 2.74)  p=0.72 | 5.3 (4.5) | 0.20 (-0.73, 1.14)  p=0.60 |
|  | Intervention | 47.7 (8.0) |  | 5.4 (4.6) |  |
| Teachers only (n=267) | Control | 46.9 (8.9) | 0.58 (-2.21, 3.38)  p=0.61 | 5.5 (4.6) | 0.32 (-0.73, 1.37)  p=0.47 |
|  | Intervention | 47.8 (8.3) |  | 5.5 (4.5) |  |

*Notes*

1. Adjusted for baseline wellbeing score and FSM
2. Adjusted for baseline PHQ-9 score and FSM

Table 2 shows the results for the student outcomes. Once baseline score, school year and FSM were adjusted for, the intervention group had a higher mean wellbeing score at follow up and a lower mean difficulties score compared to the control group.

Table 2: comparison of mean wellbeing scores for students at follow up in intervention and control arms, adjusted for baseline wellbeing, FSM and school year

|  | Wellbeing (n=1524) | | Difficulties (n=1484) | |
| --- | --- | --- | --- | --- |
|  | Mean WEMWBS score (SD) | Difference  (95% CIs)^1^ | Mean  SDQ score (SD) | Difference  (95% CIs)^2^ |
| Control | 45.8 (9.8) | 0.64 (-0.22, 1.49)  p=0.12 | 13.2 (6.1) | -1.37 (-2.07, -0.67)  p<0.01 |
| Intervention | 48.1 (9.6) |  | 11.2 (6.0) |  |

*Notes*

1. Adjusted for baseline wellbeing, FSM and school year
2. Adjusted for baseline difficulty, FSM and school year

References

[1] Kahan BC, Jairath V, Doré CJ, Morris TP: The risks and rewards of covariate adjustment in randomized trials: an assessment of 12 outcomes from 8 studies. *Trials,* 2014, **15**:139.

[2] Van Buuren S, Boshuizen HC, Knook DL: Multiple imputation of missing blood pressure covariates in survival analysis. *Statistics in Medicine*, 1999, **18**:681-694.
